# A berberine-loaded hydrogel for the treatment of atopic dermatitis through antibacterial activity, inhibition of inflammation and modulation of oxidative stress

**DOI:** 10.3389/fimmu.2026.1740911

**Published:** 2026-01-21

**Authors:** Ze Peng, Xin-Yu Wei, Xin Zeng, Gulinigaer anwaier, Xun Zhou, Bing-Jun Shi

**Affiliations:** 1 Department of Dermatology, Chongqing Traditional Chinese Medicine Hospital, Chongqing, China; 2 Chongqing Key Laboratory of Traditional Chinese Medicine for Prevention and Cure of Metabolic Diseases, Chongqing University of Chinese Medicine, Chongqing, China

**Keywords:** atopic dermatitis, berberine, hydrogel, inflammation, oxidative stress

## Abstract

**Background:**

Atopic dermatitis (AD) is a chronic, pruritic, immune-mediated inflammatory skin disorder characterized by Th2-dominant immune response, which may contribute to systemic inflammation. Hydrogels, as drug delivery vehicles, demonstrate excellent biocompatibility and superior moisturizing capabilities. Berberine exhibits anti-inflammatory, antibacterial, and immunomodulatory properties. However, the therapeutic efficacy and underlying mechanisms of berberine-loaded hydrogel (BHG) in the management of AD remain insufficiently elucidated.

**Methods:**

The morphology and structure of the hydrogel were examined using scanning electron microscopy. *In vitro* biocompatibility of the BHG was assessed via the CCK-8 assay and hemolysis testing. *In vivo*, an AD model was induced in mice by topical application of DNFB to the shaved dorsal skin. Clinical symptoms, including erythema, edema, and crusting, were monitored, and serum levels of IL-4, IL-13, IgE, and histamine were quantified using ELISA. H&E staining was performed to evaluate epidermal and dermal thickness, while toluidine blue staining was employed to assess mast cell infiltration in the dermis. Immunohistochemical staining was conducted to examine the expression of skin barrier-related proteins, and immunofluorescence staining was utilized to detect reactive oxygen species (ROS) levels in skin tissues. The expression levels of PI3K, AKT, and NF-κB pathway proteins were analyzed by Western blotting.

**Results:**

The BHG exhibited no adverse effect on HaCaT cell viability and demonstrated effective inhibition of Staphylococcus aureus proliferation. It significantly alleviated dermatological symptoms in AD mice, including erythema, edema, and crusting, while reducing scratching frequency, epidermal thickness, and mast cell infiltration. The formulation suppressed the expression of Th2-associated cytokines, including IL-4, IL-13, TNF-α, IL-6, and IgE. In the dorsal skin of AD mice, the BHG reduced levels of ROS and malondialdehyde (MDA), while increasing superoxide dismutase (SOD) activity. Furthermore, it enhanced the expression of skin barrier proteins such as filaggrin, occludin, and ZO-1, and downregulated the expression of phosphorylated PI3K, AKT, and NF-κB (p-PI3K, p-AKT, and p-NF-κB) proteins.

**Conclusion:**

The BHG exhibits favorable biocompatibility and potent antibacterial activity, and is capable of restoring the skin barrier and ameliorating dermatological symptoms in AD mice. Its anti-inflammatory and antioxidant effects may be mediated through modulation of the PI3K/AKT/NF-κB signaling pathway.

## 1 Introduction

Atopic dermatitis (AD) is a chronic and heterogeneous skin disease driven by genetic, immune, and environmental factors. It is projected that by 2050, there will be 148 million cases of AD worldwide, with an estimated prevalence of 15–25% in children and 3–7% in adults (1, 2). Key features of AD include an eczematous rash accompanied by intense pruritus, which can significantly impair patients’ quality of life. The pathogenesis of AD involves a complex interplay between epidermal barrier dysfunction and immune dysregulation characterized by Th2 (3). Genetically predisposed individuals exhibit impaired skin barrier function, leading to increased transepidermal water loss and elevated skin permeability, resulting in xerosis and desquamation. This compromised barrier facilitates the penetration of allergens and promotes colonization by microorganisms such as Staphylococcus aureus and Malassezia spp., triggering aberrant immune activation and inflammatory responses that manifest as eczematous lesions and pruritus. Repetitive scratching and excessive washing further exacerbate skin inflammation through mechanical damage and skin barrier disruption. Therefore, therapeutic strategies for AD primarily focus on restoring epidermal barrier integrity, modulating cutaneous microbiota, and suppressing inflammation (4). For decades, long-term disease control has remained challenging due to limited treatment options, which largely consist of topical and systemic immunosuppressive agents (2). Hence, there is a pressing clinical need for novel, safe, and effective therapies to improve outcomes in AD.

Berberine, a quaternary ammonium alkaloid derived from the traditional Chinese medicinal herb Coptidis rhizoma, is the principal bioactive constituent with potent antibacterial and anti-inflammatory properties (5–7). Berberine exerts anti-methicillin-resistant Staphylococcus aureus (MRSA) effects by targeting and inhibiting the biosynthesis pathway of wall teichoic acid (8). Additionally, berberine effectively suppresses the production of pro-inflammatory cytokines, including TNF-α, IL-1β, and IL-6, thereby attenuating the inflammatory cascade (5, 9). It also enhances the expression of anti-inflammatory mediators such as IL-10 and TGF-β, contributing to immune homeostasis (10). Furthermore, berberine reduces ROS generation and lipid peroxidation while increasing the activity of antioxidant enzymes such as SOD and catalase (CAT), thereby mitigating oxidative stress and cellular damage (11, 12). It has been shown to enhance host immunity and modulate systemic immune responses (13). Given its multifaceted antibacterial, anti-inflammatory, and antioxidant activities, berberine holds promise as an adjuvant agent in the clinical management of AD. However, its low *in vivo* bioavailability limits its therapeutic potential, highlighting the urgent need for innovative topical delivery systems.

**Figure 1:**
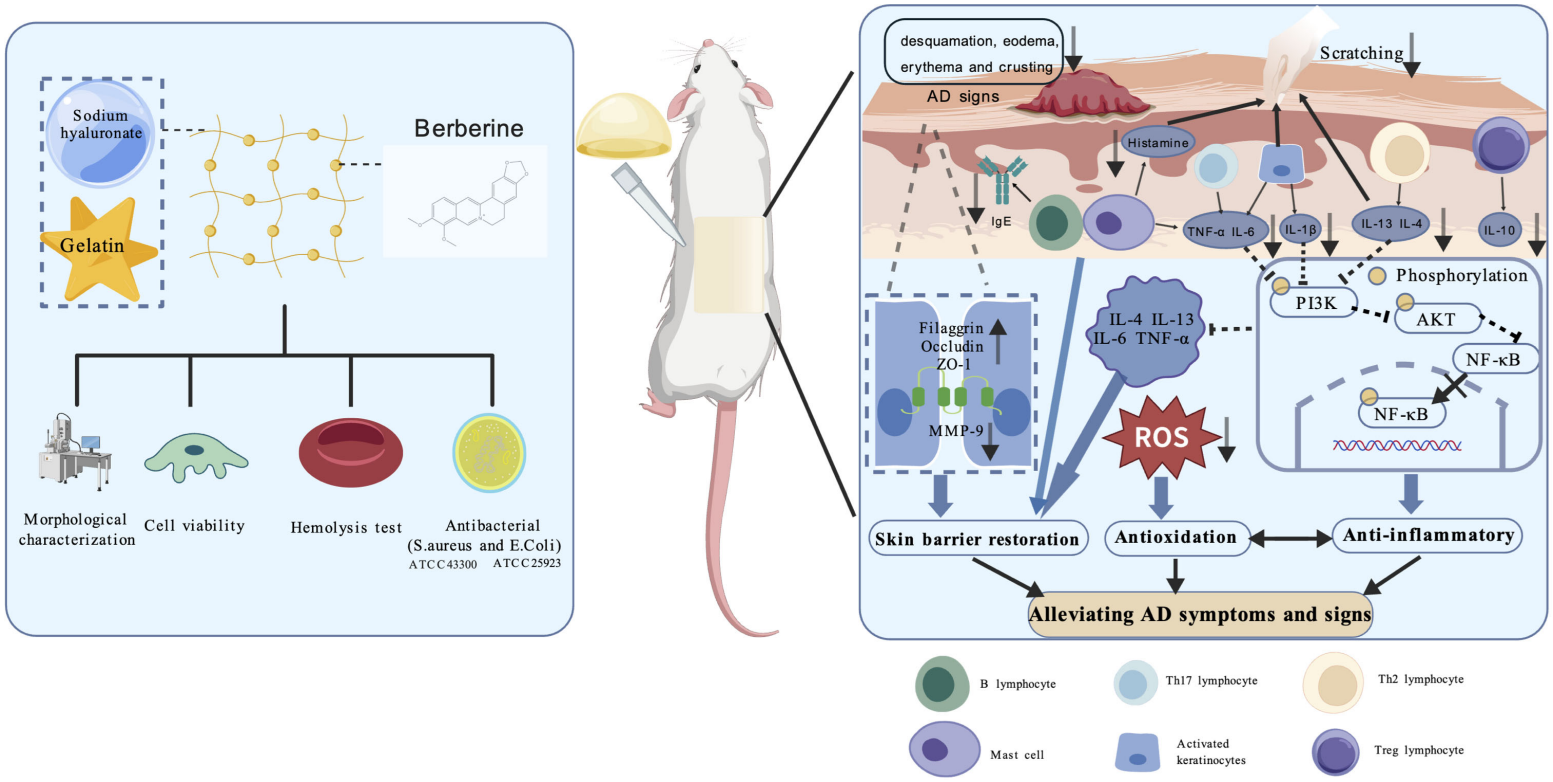
Schematic illustration of the therapeutic mechanism of BHG in alleviating symptoms of atopic dermatitis.

Conventional AD management relies on emollients or creams to maintain skin hydration or deliver therapeutics, yet these formulations often provide only transient epidermal barrier protection and short drug residence times (14). In recent years, hydrogel-based therapies have garnered growing interest among clinicians for the treatment of AD. Hydrogels are considered promising candidates for dermal applications due to their excellent biocompatibility, sustained drug release profiles, and superior moisturizing properties (15–17). In this study, we developed a BHG system using sodium hyaluronate and gelatin to enable synergistic antibacterial, anti-inflammatory, and antioxidant effects for AD therapy. We further evaluated the *in vitro* safety and antimicrobial efficacy of the formulation, as well as its *in vivo* therapeutic potential in an AD mouse model, demonstrating enhancing skin barrier proteins, ROS scavenging, and suppression of Th2-associated inflammatory cytokines (**Figure 1**). **Figure 1** was created with BioGDP.com (18).

## 2 Materials and methods

### 2.1 Chemical reagents and antibodies

All chemicals were purchased from Sigma-Aldrich (St. Louis, MO, USA) unless otherwise specified. Enzyme-linked immunosorbent assay (ELISA) kits were obtained from Enzyme Immunoassay Co., Ltd. (Jiangsu, China). The Calcein/PI Live/Dead Viability/Cytotoxicity Assay Kit, Cell Counting Kit-8 (CCK-8), and ROS detection kit were acquired from Beyotime Biotechnology Co., Ltd. (Shanghai, China). PCR reagents were provided by Accurate Biotechnology Co., Ltd. (Changsha, China). CAT, MDA, SOD, glutathione (GSH), and nicotinamide adenine dinucleotide (NADH) assay kits were purchased from Beyotime Biotechnology Co., Ltd. (Shanghai, China). Anti-PI3K (AF6241) and anti-phospho-PI3K (AF3242) antibodies were sourced from Affinity Biological Research Center (Jiangsu, China). Anti-β-actin (66009-1), anti-NF-κB p65 (66535-1), anti-phospho-NF-κB p65 (82335-1), anti-AKT (10176-2), and anti-phospho-AKT (66444-1-Ig) antibodies were obtained from Proteintech Group, Inc. (Wuhan, China). Staphylococcus aureus (S. aureus, ATCC 43300) and Escherichia coli (E. coli, ATCC 25923) were obtained from the Institute of Burn Research, Southwest Hospital, Third Military Medical University.

### 2.2 Animal

Fifty female BALB/c mice, aged 6–8 weeks, were used in this study. After one week of acclimatization, the animals were randomly assigned to five experimental groups: control group, AD model group, blank hydrogel group (HG), BHG group, and dexamethasone group (Dex). One day prior to the experiment, hair was removed from a 2 cm × 3 cm area on the dorsal skin of each mouse using an electric shaver followed by depilatory cream. The control group received vehicle solution of the 2,4-dinitrofluorobenzene DNFB inducer (a 4:1 acetone/olive oil mixture). On days 1 and 3 of the experiment, 100 μL of 0.5% DNFB solution was administered via micropipette and evenly applied to the dorsal skin, with an additional 20 μL applied to the ears. Starting on day 5, mice were treated every other day with 0.2% DNFB solution, while HG, BHG, and Dex administrations were initiated concurrently and administered once daily for two consecutive weeks. Successful induction of the AD model was confirmed by the presence of clinical signs including erythema, mild erosion, edema, crusting, and increased scratching frequency. All animal procedures were conducted in accordance with the guidelines approved by the Institutional Animal Care and Use Committee of Chongqing Medical University (IACUC-CQMU) (approval number: IACUC-CQMU-2024–0579).

### 2.3 Preparation of BHG

Equal volumes of 1% sodium hyaluronate solution and 10% gelatin solution were mixed and stirred in a 55°C water bath to form a homogeneous composite hydrogel matrix. Berberine (40 mg/mL) was added proportionally to the hydrogel and continuously stirred under the same temperature conditions, followed by repeated freeze-thaw cycles until complete incorporation and uniform distribution of berberine within the hydrogel matrix were achieved.

### 2.4 Characterization of hydrogels

The morphology and pore size of the hydrogel were examined using scanning electron microscopy (HITACHI SU-X650, Tokyo, Japan). The hydrogel was freeze-dried under vacuum for 48 hours prior to analysis. Following gold sputter coating, the sample was imaged under scanning electron microscopy (SEM).

### 2.5 Antimicrobial activity

To evaluate the antimicrobial activity of the hydrogel, samples were aseptically treated with UV irradiation and subsequently incubated with Staphylococcus aureus and Escherichia coli. Bacterial cultures of S. aureus and E. coli were grown in Luria-Bertani (LB) broth and maintained at 37°C under shaking conditions at 160 rpm. A bacterial suspension with an initial concentration of 10^6^ CFU/mL was incubated with the hydrogel for 12 hours, after which the inhibition rate was determined using a microplate reader (BioTek, USA) by measuring optical density at 600 nm. Additionally, the bacterial suspension was adjusted to an OD_600_ of approximately 0.6 before being inoculated into sterile LB liquid medium. Sterile hydrogel specimens were introduced into test tubes containing 5 mL of the bacterial suspension and incubated at 37°C for 12 hours. Subsequently, 10 μL of the resuspended culture was plated onto solid agar medium, followed by incubation at 37°C for 12 hours to assess antibacterial efficacy. The resulting colonies were documented by photography.

### 2.6 *In vitro* safety evaluation of hydrogels

The HaCaT cell line was purchased from Hysigen Bio. Cells were cultured in high-glucose DMEM supplemented with 10% fetal bovine serum and 1% penicillin-streptomycin at 37°C in a humidified atmosphere containing 5% CO_2_. The BHG was evenly spread onto the bottom of 96-well or 24-well plates and sterilized by UV irradiation for 12 hours prior to use.

To evaluate the effect of the hydrogel on HaCaT cell viability and proliferation, cells were seeded at a density of 6,000 cells per well in 96-well plates and incubated for 24 hours. Subsequently, the culture supernatant was replaced with fresh medium containing 10% CCK-8 solution, and the cells were further incubated for 2 hours. Then, 100 μL of the resulting solution was transferred to a new 96-well plate, and the optical density (OD) was measured at 450 nm using a microplate reader (BioTek, USA). For fluorescence live/dead staining, cells were seeded at a density of 10,000 cells per well in 24-well plates and incubated for 24 hours. After one wash with phosphate-buffered saline (PBS), 250 μL of working solution containing calcein acetoxymethyl ester (Calcein AM) and propidium iodide (PI) was added to each well and incubated at 37°C for 30 minutes in the dark. Fluorescence images were captured using a fluorescence microscope (Leica, Germany).

To assess the *in vitro* hemocompatibility of the hydrogel, whole blood was collected from healthy mice via orbital sinus puncture. An equal volume of normal saline was added to dilute the blood, followed by centrifugation at 3,000 rpm for 10 minutes. The supernatant was discarded, and the erythrocyte pellet was washed 2–3 times with normal saline until the supernatant became colorless. A 2% (v/v) red blood cell (RBC) suspension was prepared in normal saline. Normal saline served as the negative control, and distilled water served as the positive control. Hydrogel samples were cut into 5 × 5 mm² pieces, thoroughly mixed with the RBC suspension, and incubated in a constant-temperature water bath at 37°C for 1 hour. After centrifugation under the same conditions, the appearance of the supernatant in each tube was visually inspected, and 100 μL of the supernatant was transferred to a 96-well plate for OD measurement at 540 nm.

### 2.7 Dermatitis score

The severity of AD lesions was clinically assessed based on four parameters: edema, erythema, erosion, and scaling or dryness. Severe manifestations were assigned a score of 3, moderate lesions a score of 2, mild lesions a score of 1, and absence of lesions was scored as 0. The total dermatitis score for each subject ranged from 0 to 12. Prior to the end of the experiment, spontaneous scratching behavior was observed and recorded over a 10-minute period, with continuous scratching episodes counted as a single event.

### 2.8 Pathological staining

At the conclusion of the experiment, heart, liver, spleen, kidney, dorsal skin, and ears were harvested from mice and fixed in 4% paraformaldehyde for 24 hours. Subsequently, paraffin-embedded tissue blocks were sectioned at a thickness of 4 μm using a microtome. Following deparaffinization and rehydration, the sections were stained with Hematoxylin-eosin (H&E), and histological architecture and cellular morphology were examined under a light microscope (Leica, Germany).

To evaluate mast cell infiltration in skin tissues, sections were stained with 0.25% toluidine blue solution for 20 minutes. The number of positively stained mast cells (appearing dark purple-blue) was quantified by light microscopy.

### 2.9 Immunohistochemistry

The tissue sections were immersed in antigen retrieval solution at 95°C for 20 minutes, then rinsed with phosphate-buffered saline (PBS). Subsequently, 5% normal serum blocking solution was applied dropwise and incubated at room temperature for 30 minutes to minimize nonspecific binding. Primary antibodies against Filaggrin, Occludin, ZO-1, and MMP-9, appropriately diluted in buffer, were added dropwise and incubated overnight at 4°C. After thorough washing with PBS, biotin-conjugated secondary antibodies were applied dropwise and incubated for 30 minutes at room temperature. Next, horseradish peroxidase (HRP)-labeled streptavidin was added dropwise and incubated for an additional 30 minutes at room temperature. Thereafter, DAB (3,3′-diaminobenzidine) chromogen solution was applied, and the slides were monitored under a microscope for color development. The reaction was terminated by rinsing with distilled water when distinct positive staining was observed. Finally, nuclei were counterstained with hematoxylin, and the tissue sections were dehydrated, cleared, and mounted for examination under a light microscope.

### 2.10 Oxidative stress levels

Fresh skin lesion tissues were used to prepare frozen sections. A working solution of the fluorescent probe DCFH-DA (10 μM) was applied dropwise to the tissue sections and incubated in the dark at 37°C for 30 minutes. After washing to remove excess probe, the slides were mounted with a DAPI-containing mounting medium and sealed. Fluorescence intensity was then visualized and recorded using a fluorescence microscope.

The frozen skin lesion samples were weighed, and the levels of CAT, MDA, SOD, GSH, and the NAD^+^/NADH ratio were quantitatively determined according to the manufacturer’s instructions for the respective assay kits.

### 2.11 Western blotting

Skin tissue samples were homogenized, lysed, and centrifuged to collect the supernatant. Protein concentration was determined using the BCA assay. Protein samples were prepared by addition of protein loading buffer, boiled for 10 minutes, and stored at -80°C. Equal amounts of protein were loaded and separated by sodium dodecyl sulfate-polyacrylamide gel electrophoresis (SDS-PAGE), followed by transfer onto polyvinylidene difluoride (PVDF) membranes. Membranes were blocked with 5% non-fat milk in Tris-buffered saline containing 0.1% Tween-20 (TBST) for 2 hours at room temperature (RT). Thereafter, membranes were incubated overnight at 4°C with appropriate primary antibodies to ensure specific binding to target proteins. On the following day, membranes were incubated with horseradish peroxidase (HRP)-conjugated secondary antibodies at room temperature. Protein bands were visualized using an enhanced chemiluminescence detection system (Bio-Rad, USA), and band intensities were quantified by densitometry using ImageJ software.

### 2.12 Quantitative real-time PCR

Total RNA was extracted from dorsal skin lesion tissues using the TRIzol reagent method, and RNA concentration and purity were assessed with a NanoDrop spectrophotometer. The extracted RNA was reverse transcribed into complementary DNA (cDNA) using a reverse transcription kit according to the manufacturer’s instructions. Quantitative real-time PCR (qRT-PCR) was performed using SYBR^®^ Green Pro Taq HS Premix II on a Bio-Rad CFX96 Real-Time System (Bio-Rad Laboratories, USA). The relative mRNA expression levels were calculated using the 2^-^ΔΔC_T_ method and normalized to the expression of the housekeeping gene Gapdh. Primer sequences used in this study are provided in **Supplementary Table S1**.

### 2.13 Network pharmacology

Disease-related targets were retrieved from public databases including GeneCards, OMIM, and DisGeNET. Berberine-associated targets were collected from ChEMBL, CTD, and PubChem. Target genes were then standardized using UniProt, and non-protein-coding or unverified entries were removed to ensure data accuracy. The overlapping targets between berberine and AD were identified through cross-referencing, and a Venn diagram was generated using R software to visualize the intersection. To further investigate the protein-protein interaction (PPI) network underlying the therapeutic mechanism, the shared target genes were uploaded to the STRING database for PPI network construction. Subsequently, Gene Ontology (GO) enrichment analysis and Kyoto Encyclopedia of Genes and Genomes (KEGG) pathway analysis were performed on the intersected gene set. The results were visualized to predict the potential molecular mechanisms by which berberine exerts therapeutic effects in AD.

### 2.14 Data analysis

Data were analyzed using GraphPad Prism (version 9.5.0), and one-way ANOVA was employed to assess differences among groups. All data are presented as mean ± standard error of the mean (SEM). Statistical significance was considered at *P* < 0.05.

## 3 Results

### 3.1 Safety and antibacterial properties of BHG

**Figure 2:**
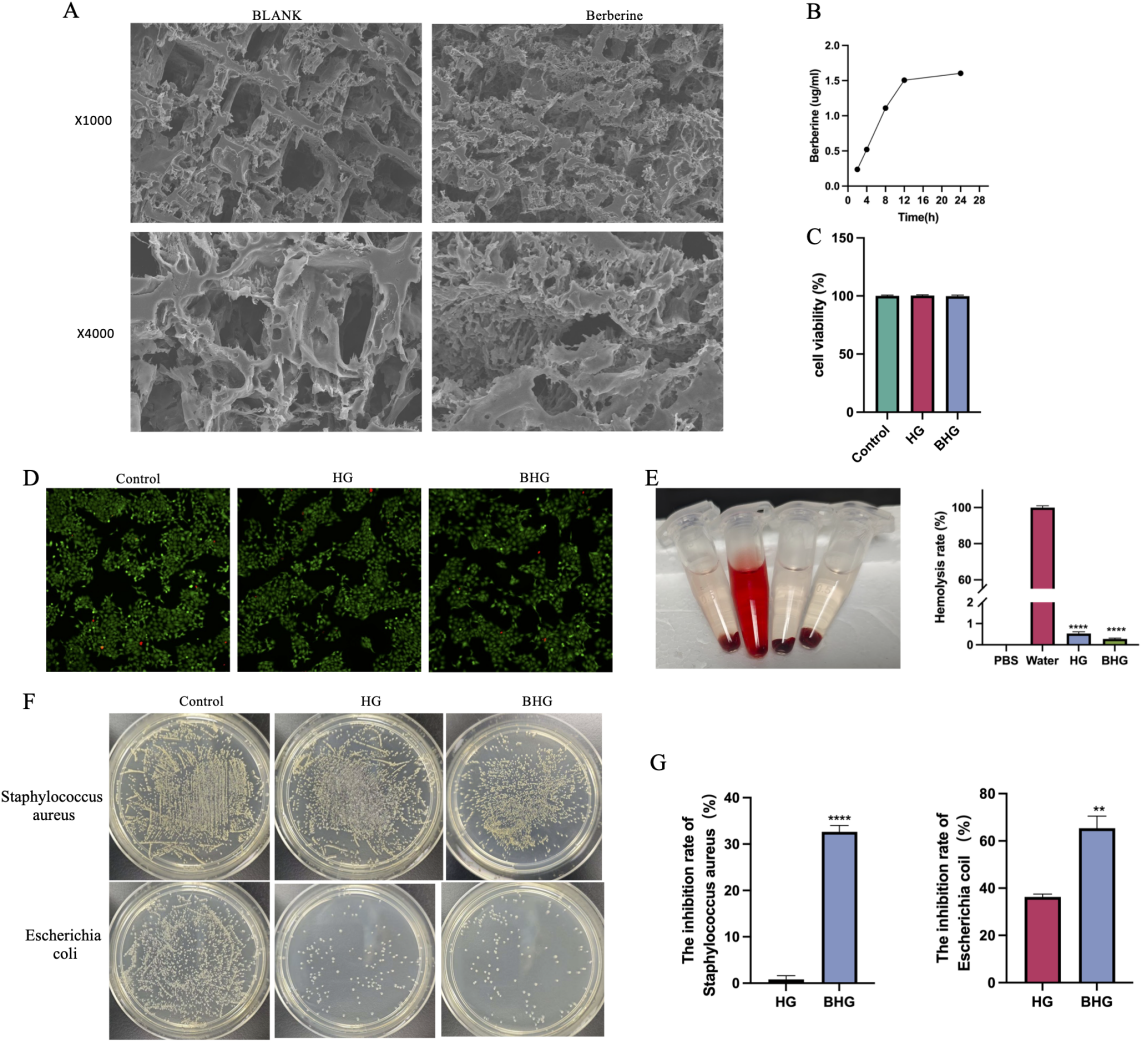
Characterization of BHG. **(A)** Scanning electron microscopy images of the hydrogel. **(B)** Transdermal delivery kinetics of berberine at different time points. **(C)** Cell viability of HaCaT cells after 24-hour culture, as assessed by CCK-8 assay. **(D)** Representative fluorescence images of Calcein AM/PI staining in HaCaT cells (scale bar = 100 μm). **(E)** Hemolysis test and calculated hemolysis rate. **(F)** Representative images showing the inhibitory effect of the hydrogel on Staphylococcus aureus and Escherichia coli. **(G)** Quantitative analysis of inhibition rates against Staphylococcus aureus and Escherichia coli. HG, Hydrogel; BHG, Berberine-loaded hydrogel. n = 3. ^**^P < 0.01, ****P < 0.0001.

Hydrogels are a class of drug delivery systems characterized by excellent water retention capacity and biocompatibility. Berberine was uniformly incorporated into the hydrogel matrix, and the structural morphology was examined using SEM. As shown in **Figure 2A**, both the blank and BHG exhibited a well-defined three-dimensional porous network structure. At 12 hours, the transdermal concentration of berberine reached 1.5 μg/mL and remained stable up to 24 hours (**Figure 2B**), demonstrating sustained release properties. Chromatograms of berberine standards, blank hydrogels, and BHGs are presented in **Supplementary Figure S1**.

To evaluate the safety profile of the BHG, cell viability assays using HaCaT keratinocytes and erythrocyte hemolysis tests were performed. CCK-8 assay results showed no significant reduction in HaCaT cell viability after exposure to the hydrogel (**Figure 2C**). Consistently, live/dead staining using Calcein AM and PI revealed minimal cell death across all treatment groups (**Figure 2D**), further confirming low cytotoxicity. In the *in vivo* experiments, H&E staining results indicated that the morphological and structural features of the liver, heart, kidney, and spleen of mice in each group were generally consistent. These results are presented in **Supplementary Figure S2**.

In addition, hemolysis testing demonstrated that the BHG induced a hemolysis rate of 0.28 ± 0.09% (n = 4), significantly lower than that of the positive control (>95%) and well below the 2% threshold for non-hemolytic biomaterials. Compared with the blank hydrogel (0.53 ± 0.2%), berberine incorporation did not significantly increase hemolytic potential, indicating favorable blood compatibility (**Figure 2E**).

*In vitro* antibacterial assays revealed that the BHG exerted inhibitory effects against both Staphylococcus aureus and Escherichia coli. The antimicrobial activity was more pronounced against Escherichia coli (**Figures 2F, G**), suggesting broad-spectrum antibacterial functionality.

### 3.2 BHG alleviates dermatitis in a mouse model of AD

**Figure 3:**
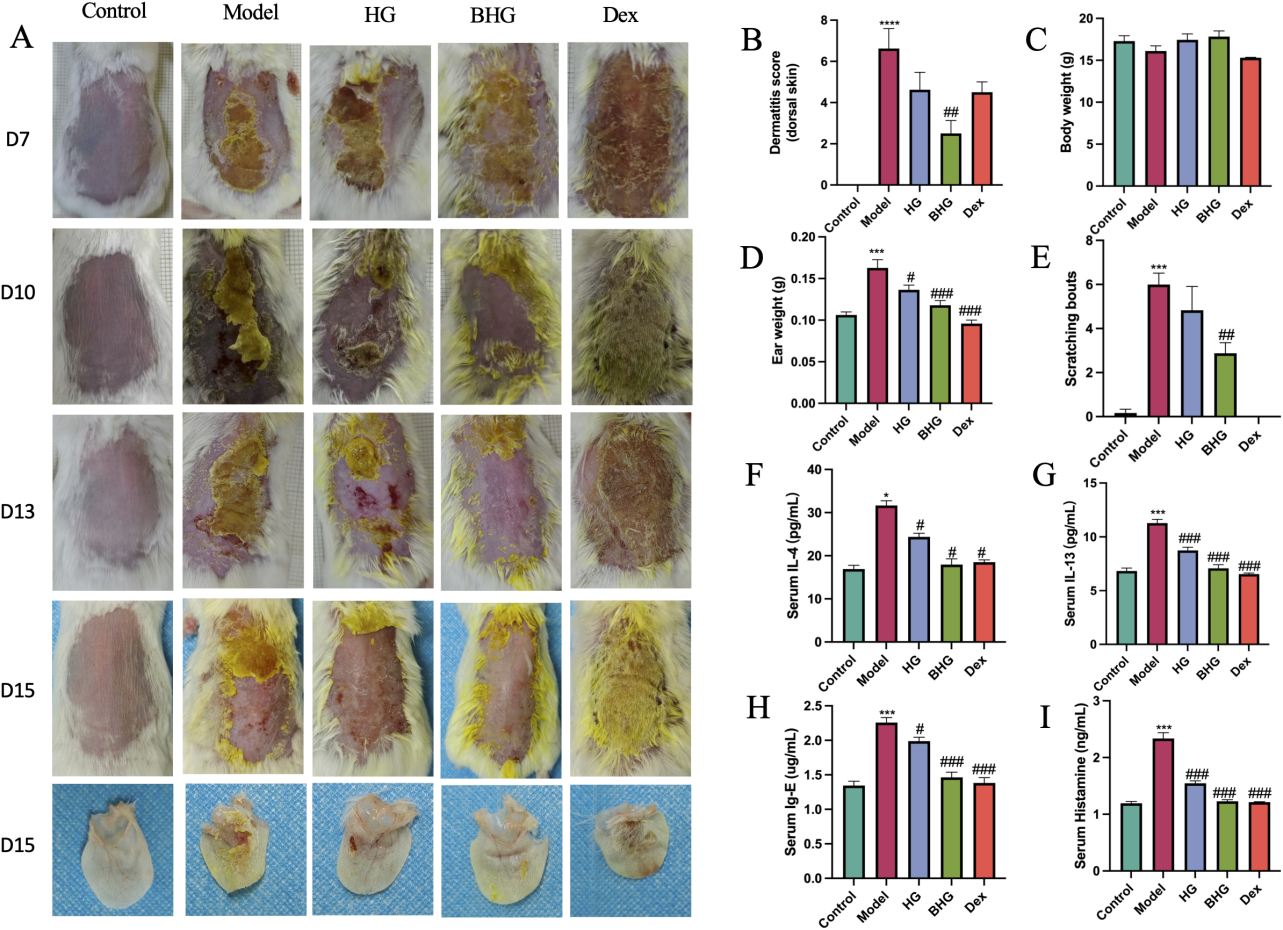
BHG alleviates symptoms of atopic dermatitis. **(A)** Representative photographs of dorsal skin and ears in each experimental group. **(B)** Dermatitis severity score, **(C)** body weight, **(D)** ear thickness (expressed as ear weight), and **(E)** scratching frequency over a defined period. Serum levels of IL-4 **(F)**, IL-13 **(G)**, IgE **(H)**, and histamine **(I)** in mice. AD, Atopic dermatitis; HG, Hydrogel; BHG, Berberine-loaded hydrogel; Dex, Dexamethasone. n=6, Compared with the control group, ^*^P < 0.05, ***P < 0.001, ^****^P < 0.0001; compared with the model group, ^#^*P* < 0.05, ^##^*P* < 0.01, ^###^*P* < 0.001, ^###^*P* < 0.0001.

A mouse model of AD induced by DNFB exhibited characteristic symptoms including skin erythema, scaling, exudation, and epidermal thickening. Starting on day 5, the BHG was applied topically to the dorsal and auricular skin of mice to evaluate its therapeutic effects on dermatitis. Mice in the model group developed severe crust formation, scaling, and erosion on both dorsal and ear skin. In contrast, dermatitis symptoms in the BHG group were significantly alleviated, with a marked reduction in clinical dermatitis scores. In the Dex treatment group, extensive crusting covered with regrowing hair was observed (**Figures 3A, B**). No significant differences in body weight were observed among the groups; however, a decreasing trend was noted in both the model and Dex groups (**Figure 3C**). Compared with the control group, ear thickness and weight were significantly increased in the model group, indicating inflammatory swelling, which was markedly reduced following treatment with either BHG or dexamethasone (**Figure 3D**).

Pruritus is a hallmark symptom of atopic dermatitis; therefore, scratching behavior was recorded over a 10-minute observation period. The results showed no scratching episodes in the control or Dex groups, whereas model mice exhibited an average of six scratching bouts. Treatment with BHG reduced the average scratching frequency by approximately 50% (**Figure 3E**).

Subsequently, serum levels of key inflammatory markers were analyzed. ELISA results demonstrated that IL-4, IL-13, IgE, and histamine levels were significantly lower in both the BHG and Dex groups compared to the model group (**Figures 3F–I**), indicating effective suppression of systemic allergic inflammation.

### 3.3 BHG reduces skin thickness and mast cell infiltration in a mouse model of AD

**Figure 4:**
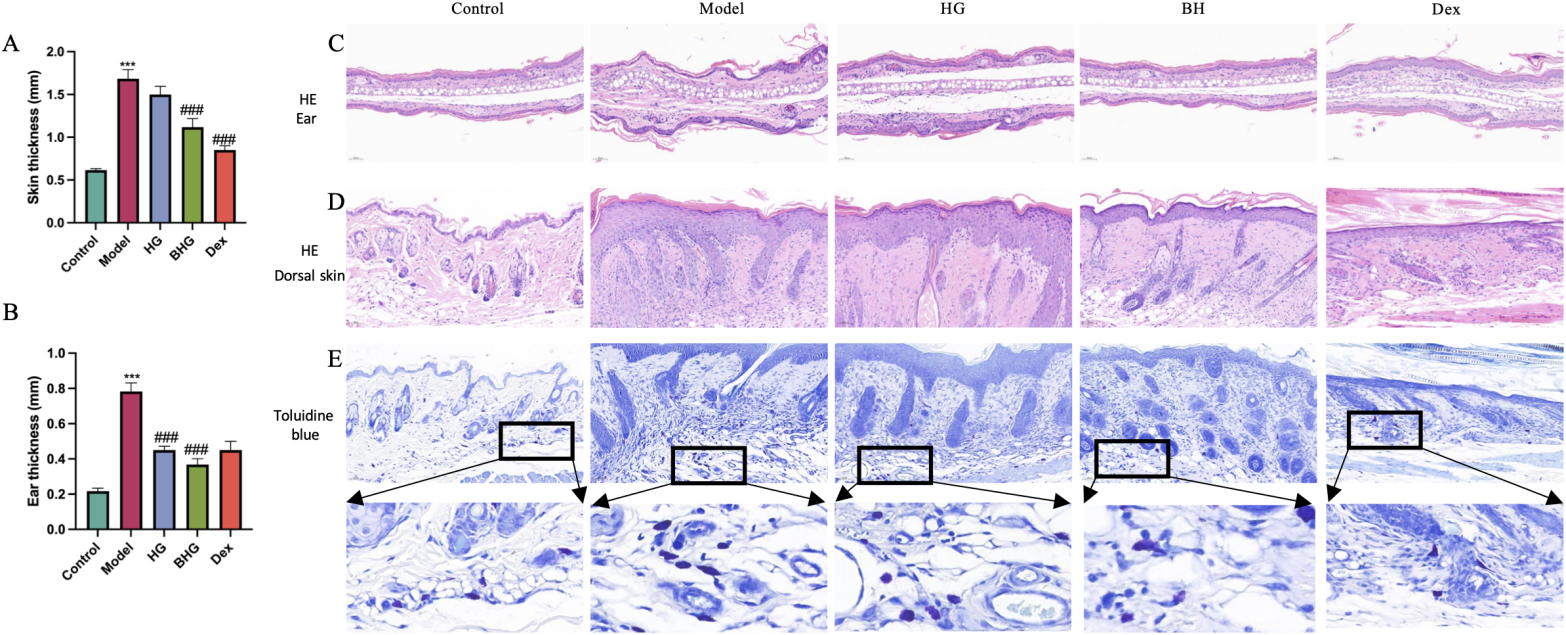
Effects of BHG on skin tissue thickness, histopathological morphology, and mast cell infiltration in mice with atopic dermatitis. **(A, B)** Measurement of dorsal skin and ear thickness using a vernier caliper. **(C, D)** Hematoxylin and eosin (H&E) staining of ear and dorsal skin tissues (scale bar = 100 μm). **(E)** Toluidine blue staining of dorsal skin sections showing mast cell distribution (scale bar = 100 μm; mast cells appear blue-purple). AD, Atopic dermatitis; HG, Hydrogel; BHG, Berberine-loaded hydrogel; Dex, Dexamethasone. n = 6. Compared with the control group, ^***^P < 0.001; compared with the model group, ^###^*P* < 0.001.

Patients with atopic dermatitis often exhibit skin thickening; therefore, dorsal and auricular skin thickness in mice was measured using a vernier caliper. Results showed that skin thickness was significantly increased in the model group, and treatment with BHG led to a significant reduction (**Figures 4A, B**). Histopathological changes in ear and dorsal skin were further evaluated by H&E staining. In both the model and blank hydrogel groups, marked epidermal and dermal thickening was observed, accompanied by hyperkeratosis and parakeratosis in the epidermis, as well as inflammatory cell infiltration in the dermis. Following treatment with BHG, skin thickness decreased and inflammatory cell infiltration was reduced. In contrast, although dexamethasone treatment reduced skin thickness, it was associated with persistent inflammatory cell infiltration and increased tissue rigidity, which may reflect adverse effects related to long-term corticosteroid use (**Figures 4C, D**).

Mast cell accumulation is closely associated with Th2-type immune responses, and represents a key immunopathological feature of atopic dermatitis. Toluidine blue staining of dorsal skin lesions revealed a significant increase in dermal mast cells in the model group, while mast cell infiltration was markedly reduced following treatment with BHG, indicating alleviation of allergic inflammation (**Figure 4E**).

### 3.4 Network pharmacology analysis results

**Figure 5:**
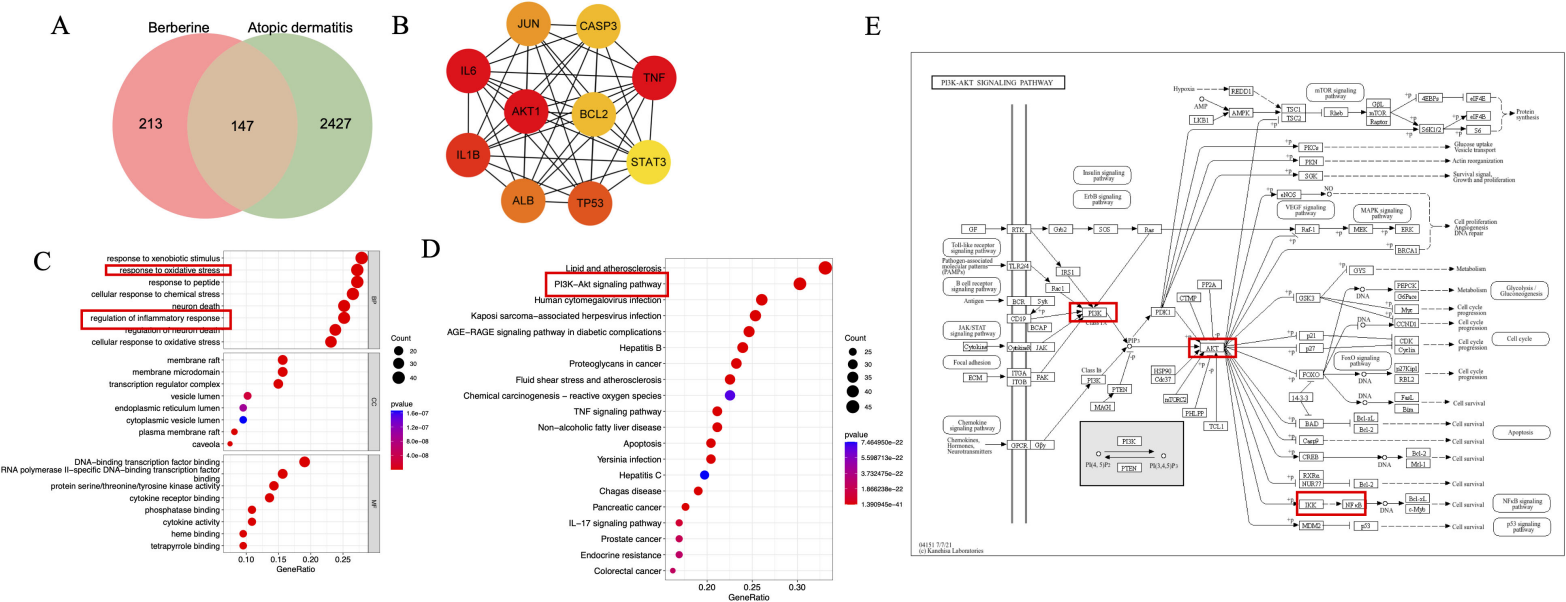
Results of network pharmacology analysis. **(A)** Venn diagram showing the intersection of target sets. **(B)** Protein-protein interaction (PPI) network. **(C)** Gene Ontology (GO) functional enrichment map. **(D)** KEGG pathway enrichment analysis. **(E)** Schematic representation of the PI3K/AKT signaling pathway and its associated targets.

A total of 147 target genes were commonly identified in both berberine and AD, with the top four being AKT1, TNF-α, IL-6, and IL-1β (**Figures 5A, B**). GO biological process enrichment analysis revealed that these shared targets were predominantly involved in oxidative stress and inflammatory response pathways (**Figure 5C**). KEGG pathway enrichment analysis indicated that the PI3K-AKT signaling pathway is a key potential mechanism underlying the therapeutic effects of berberine in AD (**Figure 5D**). Previous studies have demonstrated that the PI3K/AKT pathway regulates the activation of downstream NF-κB (19, 20). As a central transcriptional regulatory hub, the NF-κB signaling pathway promotes the expression of various pro-inflammatory mediators, including cytokines such as IL-6, TNF-α, and IL-1β, chemokines such as IL-8 and MCP-1, and matrix metalloproteinase MMP-9 (21). This pathway is critically involved in immune responses, inflammatory processes, and cell survival, highlighting its significance in the pathogenesis of AD.

### 3.5 BHG reduces oxidative stress levels in skin lesions of a mouse model of AD

**Figure 6:**
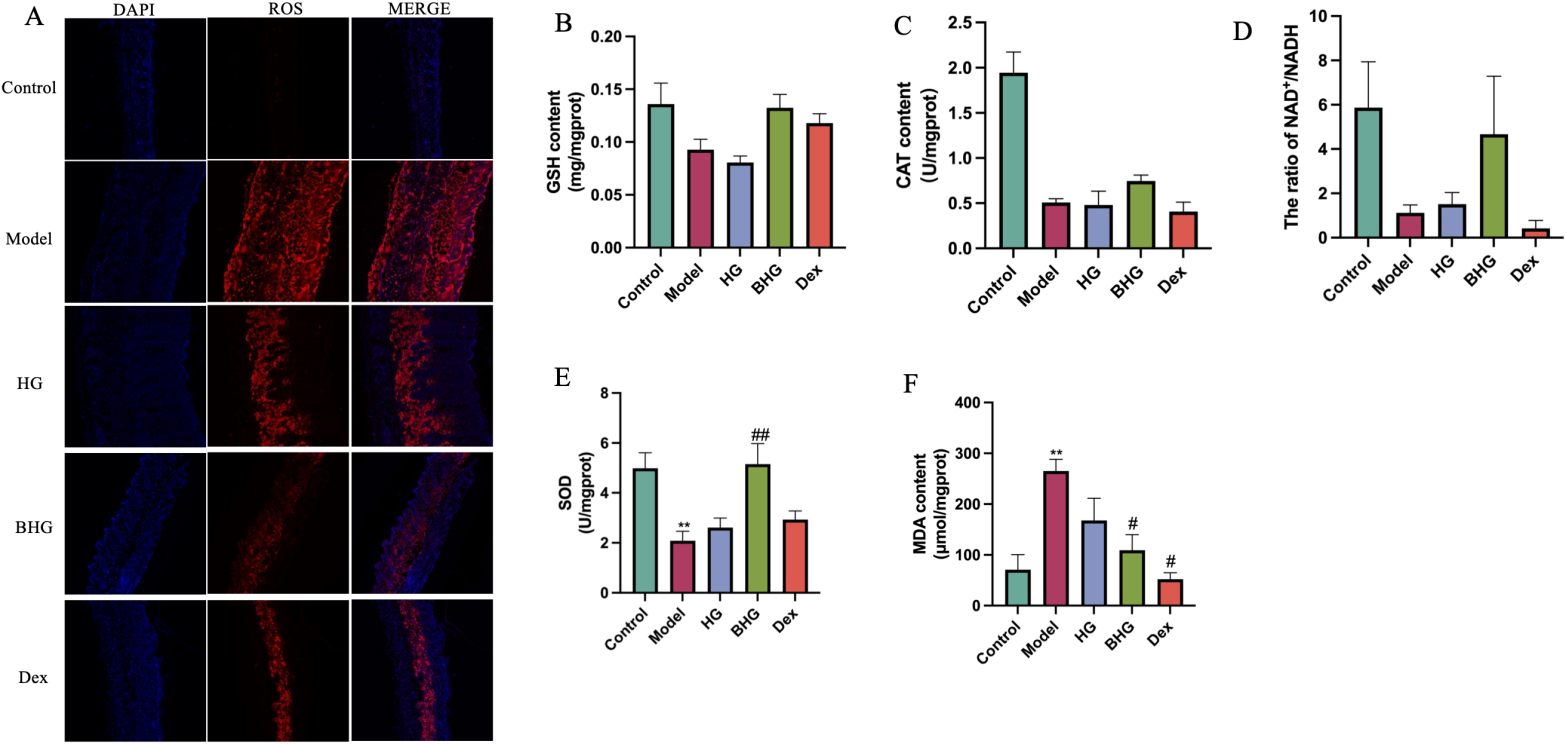
BHG reduces oxidative stress levels in mice with atopic dermatitis. **(A)** Immunofluorescence staining for reactive oxygen species (ROS) in dorsal skin tissues (scale bar = 100 μm). **(B–F)** Levels of malondialdehyde (MDA), catalase (CAT), superoxide dismutase (SOD), and NAD+/NADH ratio in dorsal skin homogenates. AD, Atopic dermatitis; HG, Hydrogel; BHG, Berberine-loaded hydrogel; Dex, Dexamethasone. n = 6. Compared with the control group, ^**^P < 0.01; compared with the model group, ^#^*P* < 0.05, ^##^*P* < 0.01.

ROS levels in dorsal skin tissues of mice were assessed using immunofluorescence staining. As shown in **Figure 6A**, ROS fluorescence intensity was significantly elevated in the AD model group compared to the control group. Fluorescence intensity decreased in both the HG and BHG groups, with the lowest ROS levels observed in the BHG group. Subsequently, the expression of oxidative stress-related enzymes was evaluated. Compared with the control group, the levels of GSH, CAT, and the NAD^+^/NADH ratio were significantly reduced in the AD model group, and treatment with BHG restored these parameters toward normal levels, although the differences did not reach statistical significance (**Figures 6B–D**). In contrast, the BHG group exhibited a significant increase in SOD activity compared to the AD model group (**Figure 6E**), along with a significant reduction in MDA levels (**Figure 6F**). These findings collectively indicate that the BHG ameliorates oxidative stress in the skin tissue of a mouse model of AD.

### 3.6 BHG enhances the expression of skin barrier proteins in a mouse model of AD

**Figure 7:**
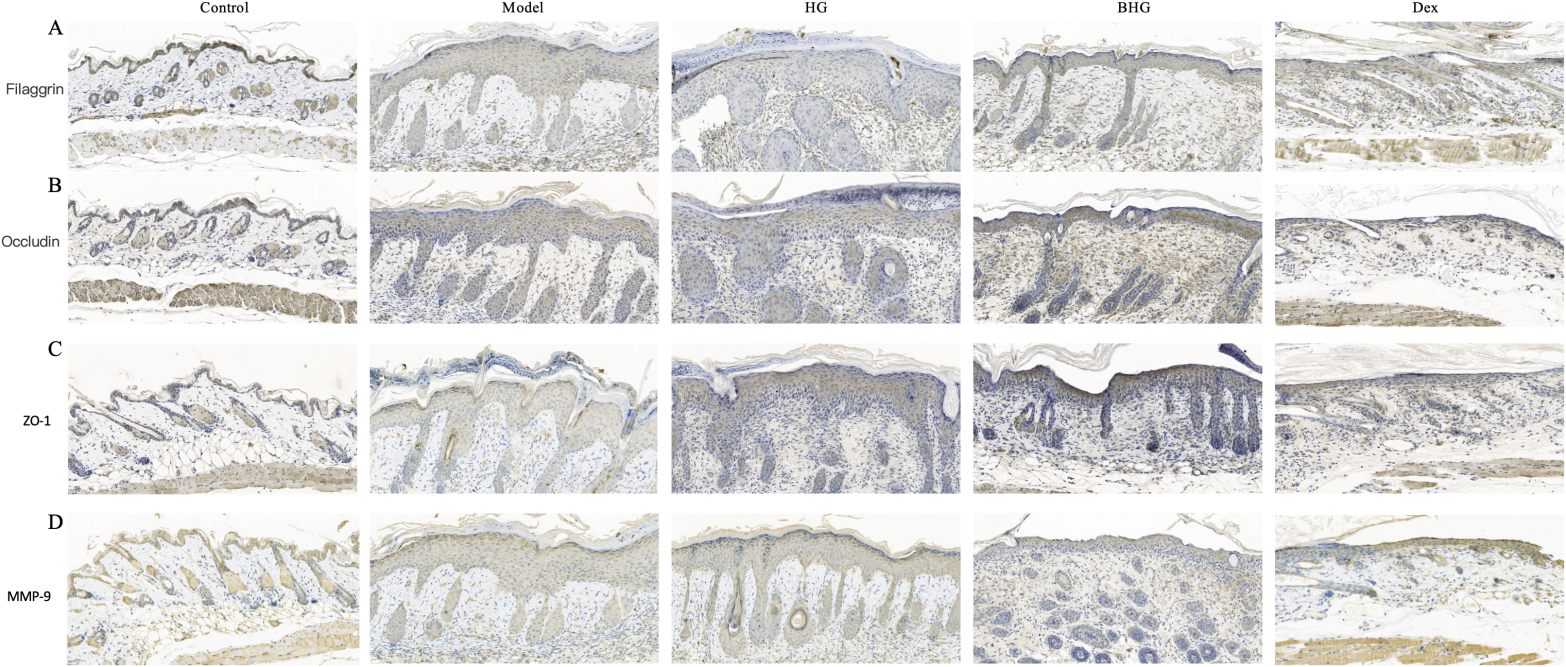
BHG restores skin barrier function in mice with atopic dermatitis. **(A–D)** Immunohistochemical staining of filaggrin, occludin, ZO-1, and MMP-9 proteins in dorsal skin tissues from each experimental group. AD, Atopic dermatitis; HG, Hydrogel; BHG, Berberine-loaded hydrogel; Dex, Dexamethasone.n=6.

Barrier dysfunction is one of the core pathogenic mechanisms of AD, contributing to a vicious cycle with disease progression. Therefore, the expression of skin barrier-related proteins was evaluated using immunohistochemical staining. As shown in **Figures 7A–C**, the model group exhibited significantly reduced staining intensity of filaggrin, occludin, and ZO-1 in the epidermis, which was markedly restored following treatment with BHG. In contrast, MMP-9 protein expression was significantly upregulated in AD mice but downregulated in the BHG group (**Figure 7D**).

### 3.7 BHG ameliorates AD via the PI3K/AKT/NF-κB signaling pathway

**Figure 8:**
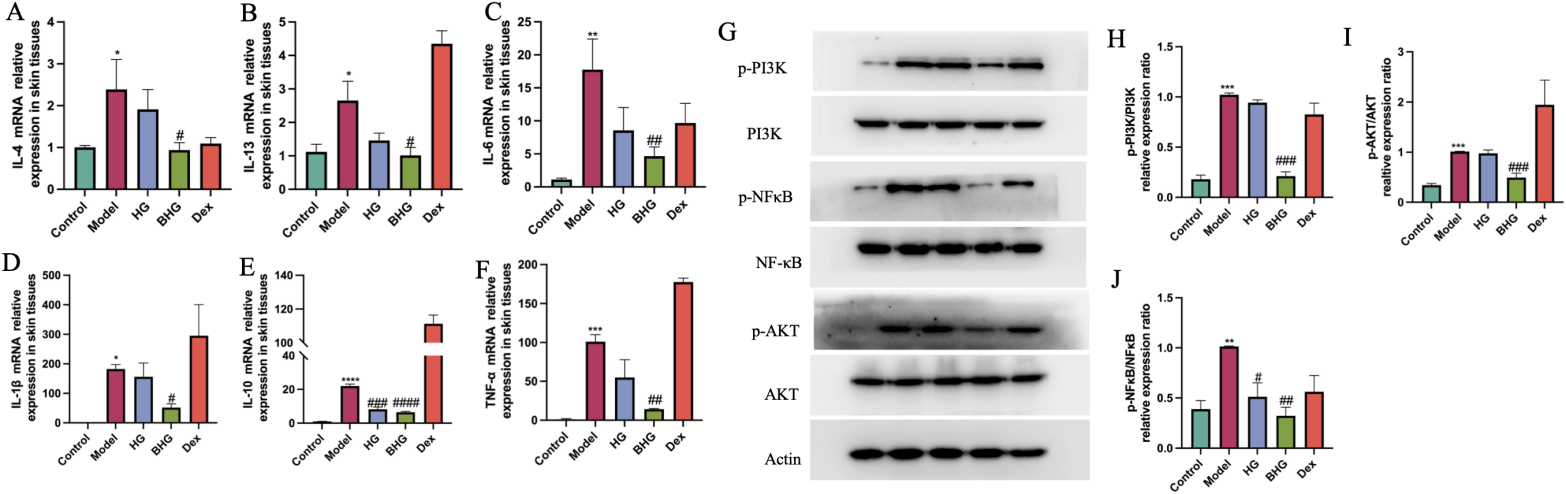
BHG reduces inflammation in mice with atopic dermatitis. **(A–F)** Relative mRNA expression levels of IL-4, IL-13, IL-6, IL-1β, IL-10, and TNF-α in dorsal skin tissues from each experimental group. **(G)** Protein expression of p-PI3K, PI3K, p-AKT, AKT, p-NF-κB, NF-κB, and β-actin in dorsal skin detected by Western blotting. **(H–J)** Quantitative analysis of protein expression levels. AD, Atopic dermatitis; HG, Hydrogel; BHG, Berberine-loaded hydrogel; Dex, Dexamethasone. n = 6. Compared with the control group, ^*^P < 0.05, ^**^P < 0.01, ***P < 0.001, ^****^P < 0.0001; compared with the model group, ^#^*P* < 0.05, ^##^*P* < 0.01, ^####^*P* < 0.0001.

Finally, the expression of inflammation-related mRNAs in dorsal skin tissue of mice was analyzed by quantitative PCR. In the model group, mRNA levels of IL-4, IL-13, IL-6, IL-1β, IL-10, and TNF-α were significantly upregulated compared to the control group. Following BHG treatment, the expression of these inflammatory factors was significantly downregulated, whereas no significant improvement was observed in the dexamethasone group (**Figures 8A–F**). Subsequently, protein expression in mouse skin tissues was assessed by Western blotting. Compared with the control group, phosphorylated PI3K, AKT, and NF-κB proteins were significantly elevated in the model group, while their expression was markedly reduced following treatment with BHG (**Figures 8G–J**).

## 4 Discussion

AD is a chronic inflammatory dermatosis closely associated with genetic predisposition and characterized by skin barrier dysfunction, microbial dysbiosis, oxidative stress, and a Th2-dominated inflammatory response. The therapeutic goals include restoration of the skin barrier, suppression of inflammation, and control of bacterial colonization. Current clinical management strategies encompass topical corticosteroids, systemic immunomodulators, phototherapy, and wet wrap therapy, which offer symptomatic relief but are limited by adverse effects such as immunosuppression, cutaneous atrophy, and the development of drug resistance (2, 22). Topical drug delivery directly targets lesional skin while minimizing systemic exposure, representing a cornerstone of AD therapy (23). However, the skin’s natural barrier function impedes effective drug penetration, and conventional emollients—such as ointments and creams—often exhibit greasy textures and potential odor, leading to suboptimal patient adherence. Therefore, there remains a critical need for the development of novel, more effective, and safer therapeutic modalities (24, 25).

With the emergence of new delivery media such as hydrogels and nanomaterials, hydrogel-based formulations have emerged as a promising alternative to traditional emollients for the management of AD, offering improved patient adherence alongside sustained therapeutic efficacy (26–28). Patients with AD frequently present with xerosis and desquamation. The high water content of hydrogels helps reduce transepidermal water loss, thereby enhancing skin hydration. Polymer components such as hyaluronic acid and chitosan mimic natural moisturizing factors and may promote keratinocyte differentiation and lipid bilayer reconstruction in the upper layers of the epidermis (15, 29). Skin lesions in AD are predominantly localized to prominent and friction-prone areas, including the face, cubital fossae, and popliteal fossae. Hydrogels provide a non-greasy, breathable matrix that adheres effectively to skin folds and curved anatomical regions without disrupting daily activities. A preference study involving 22 AD patients (30) demonstrated that hydrogels were perceived as easier to apply, suitable for use on hairy skin and across multiple body sites, and less prone to smudging compared to conventional formulations. Most participants reported greater comfort during use and no associated skin drying. Therefore, hydrogel-based therapies significantly enhance patient compliance.

Due to their favorable characteristics, including biocompatibility, bioavailability, and safety, hydrogels are widely utilized as drug delivery carriers in medical, cosmetic, and regenerative medicine applications (31). In this study, a hydrogel system was constructed using the natural polymers sodium hyaluronate and gelatin. Sodium hyaluronate provides excellent moisture retention and permeability, while gelatin serves as the structural scaffold, conferring mechanical strength and enhancing both biocompatibility and drug loading capacity. Following complete integration of berberine into the hydrogel matrix, we developed a drug-loaded hydrogel with good biocompatibility, enhanced transdermal delivery, and combined antibacterial and anti-inflammatory properties, making it suitable for topical skin application. *In vitro* studies demonstrated that the BHG had no adverse effect on the viability of HaCaT cells. *In vivo* evaluations in mice revealed no histopathological damage to the liver, kidney, heart, or other major organs, and no hemolytic activity was observed, indicating a favorable safety profile.

The IL-4 and IL-13 inflammatory pathways have been recognized as hallmark features of AD pathogenesis, playing distinct and synergistic roles in immune dysregulation, skin barrier impairment, and key clinical manifestations such as pruritus (32). In the present study, the BHG significantly reduced serum levels of inflammatory cytokines including IL-4 and IL-13, as well as their mRNA expression in dorsal skin tissue of AD mice. Pruritus remains a defining symptom of AD and constitutes a major contributor to the disease burden experienced by patients and their families (33). According to Wang’s study, in a substantial subset of AD patients, chronic pruritus is mediated through the mast cell–histamine axis, whereas elevated IgE levels are more commonly associated with acute pruritic episodes (34). We observed that the BHG markedly reduced scratching frequency within a 10-minute observation period and decreased serum levels of both IgE and histamine in AD mice. Additionally, the treatment reduced mast cell infiltration in lesional skin tissue. These findings suggest that BHGs can alleviate both acute and chronic pruritus in a mouse model of AD.

Impairment of the epithelial barrier has been shown to play a key role in the pathogenesis of AD (35, 36). Mutations in the filaggrin gene (*FLG*, *135940) have been identified as the strongest genetic risk factor for AD reported to date in both European and Asian populations (37). In this study, filaggrin expression was significantly reduced in the upper layers of the epidermis of AD mice, and both BHG and dexamethasone treatment led to a significant upregulation of filaggrin expression. *FLG* gene mutations result in deficient filaggrin synthesis, disruption of the stratum corneum architecture, increased transepidermal water loss, and impaired skin hydration (38). This barrier dysfunction creates favorable conditions for *Staphylococcus aureus* colonization. *S. aureus* colonization has been detected in the skin of 57% to 100% of children and 54% to 100% of adults with AD, with prevalence and density correlating positively with disease severity (39, 40). *In vitro*, the BHG significantly inhibited the growth of *S. aureus* and *E. coli*, demonstrating potent antibacterial activity.

In addition to filaggrin and the unique structure of the stratum corneum in the upper epidermis, tight junctions between keratinocytes also play a critical role in maintaining skin barrier integrity (41). Tight junction proteins, including occludin and ZO-1, are downregulated in the skin of patients with AD (42). Matrix metalloproteinases (MMPs), which regulate tissue remodeling in the dermis and facilitate inflammatory cell migration into the epidermis, are implicated in the pathogenesis of AD. IL-13 induces MMP-9 expression in keratinocytes, and both MMP-9 and IL-13 levels are elevated in acute AD lesions (43). Consistent with these findings, our experimental results showed that Claudin-1 and ZO-1 protein expression was downregulated, while MMP-9 protein expression was upregulated, in AD mice. This dysregulation was reversed following treatment with BHG.

As the largest organ and a critical barrier separating the body’s internal environment from the external world, the skin is continuously exposed to various exogenous agents, leading to the generation of oxidative and inflammatory mediators (44). Emerging evidence has implicated oxidative stress in the pathogenesis of AD. An imbalance in redox homeostasis has been reported in AD, particularly during disease exacerbation, characterized by elevated oxidative activity and diminished antioxidant defense capacity (45–47). Previous studies have demonstrated that berberine exhibits potent antioxidant properties in the treatment of inflammatory bowel disease, as well as cardiovascular and metabolic disorders (48, 49). In this study, the BHG significantly reduced levels of ROS and MDA, and increased SOD activity in the skin of AD mice, although changes in GSH and CAT levels were not statistically significant. These findings indicate that oxidative stress plays a pivotal role in AD progression and that berberine can mitigate oxidative stress, thereby contributing to symptom improvement.

The PI3K/AKT signaling cascade is a critical intracellular regulatory network that fundamentally governs cell proliferation, survival, metabolism, and immune response (50, 51). Dysregulated activation of this pathway has been implicated in the pathophysiology of skin cancer and immune-mediated dermatological disorders. Activation of PI3K/AKT promotes the activation of transcription factors such as NF-κB, which in turn regulate the expression of pro-inflammatory cytokines including TNF-α, IL-1β, and IL-6, thereby amplifying the inflammatory response (19, 52, 53). In patients with AD, both the PI3K/AKT and NF-κB signaling pathways exhibit aberrant activation (54, 55). Evidence indicates that suppression of NF-κB signaling can attenuate AD-associated inflammation (56, 57). To further elucidate the mechanism underlying the therapeutic effects of BHG in AD, we examined the protein expression levels of PI3K, AKT, and NF-κB, as well as the mRNA expression of TNF-α, IL-1β, and IL-6 in mouse skin tissue. Our results demonstrated that phosphorylated PI3K (p-PI3K), p-AKT, and p-NF-κB were significantly upregulated in the skin tissue of AD mice, accompanied by marked increases in mRNA levels of IL-4, IL-13, TNF-α, IL-1β, and IL-6. These alterations were reversed following topical application of BHG.

Topical glucocorticoids are commonly used as first-line anti-inflammatory agents in the management of AD. Their main adverse effect is skin atrophy, which manifests clinically as epidermal thinning, telangiectasia, spontaneous striae, fissuring, and hypertrichosis (58, 59). Therefore, long-term use of topical corticosteroids requires careful monitoring for cutaneous side effects. In our study, dexamethasone was employed as a positive control to treat AD mice. Interestingly, dexamethasone effectively reduced serum levels of IL-4, IL-13, IgE, and histamine in AD mice; however, it also induced several local adverse effects on dorsal skin, including skin thinning with atrophy and hypertrichosis. In addition, topical corticosteroids treatment has been shown to downregulate filaggrin expression, resulting in impaired barrier homeostasis and compromised integrity and cohesion of the stratum corneum (60, 61). These findings underscore the need for large-scale clinical studies to establish optimal treatment regimens in routine practice.

In summary, the BHG exhibits excellent biocompatibility and potent antibacterial activity, effectively reducing serum levels of IL-4, IL-13, IgE, and histamine in AD mice, and alleviating clinical symptoms including erythema, exudation, crusting, and pruritus. Furthermore, the BHG enhanced the expression of skin barrier proteins such as filaggrin and occludin, reduced ROS levels in lesional skin, ameliorated oxidative stress, and exerted anti-inflammatory effects via modulation of the PI3K/AKT/NF-κB signaling pathway. This study provides a novel perspective and scientific rationale for the clinical management of AD and offers new insights into the development of advanced topical therapies that not only cover the anti-inflammatory effect, but also exhibit interesting antibacterial, antioxidant, and skin barrier restorative properties for the long-term control of this chronic inflammatory dermatosis.

## Data Availability

The original contributions presented in the study are included in the article/**Supplementary Material**. Further inquiries can be directed to the corresponding author.
