# Supplementary material for: A berberine-loaded hydrogel for the treatment of atopic dermatitis through antibacterial activity, inhibition of inflammation and modulation of oxidative stress
Source: Front Immunol. 2026 Jan 21;17:1740911. doi: 10.3389/fimmu.2026.1740911 (PMC12867787; doi:10.3389/fimmu.2026.1740911)

Table S1 Primers used in RT-qPCR analysis.

Gene	Forward primer (5’-3’)	Reverse primer (5’-3’)
IL-4	GGTCTCAACCCCCAGCTAGT	GCCGATGATCTCTCTCAAGTGAT
IL-13	CAGCCTCCCCGATACCAAAAT	GCGAAACAGTTGCTTTGTGTAG
IL-6	TAGTCCTTCCTACCCCAATTTCC	TTGGTCCTTAGCCACTCCTTC
IL-1β	GCAACTGTTCCTGAACTCAACT	ATCTTTTGGGGTCCGTCAACT
IL-10	CTTACTGACTGGCATGAGGATCA	GCAGCTCTAGGAGCATGTGG
TNF-α	CTGAACTTCGGGGTGATCGG	GGCTTGTCACTCGAATTTTGAGA

Figure S1

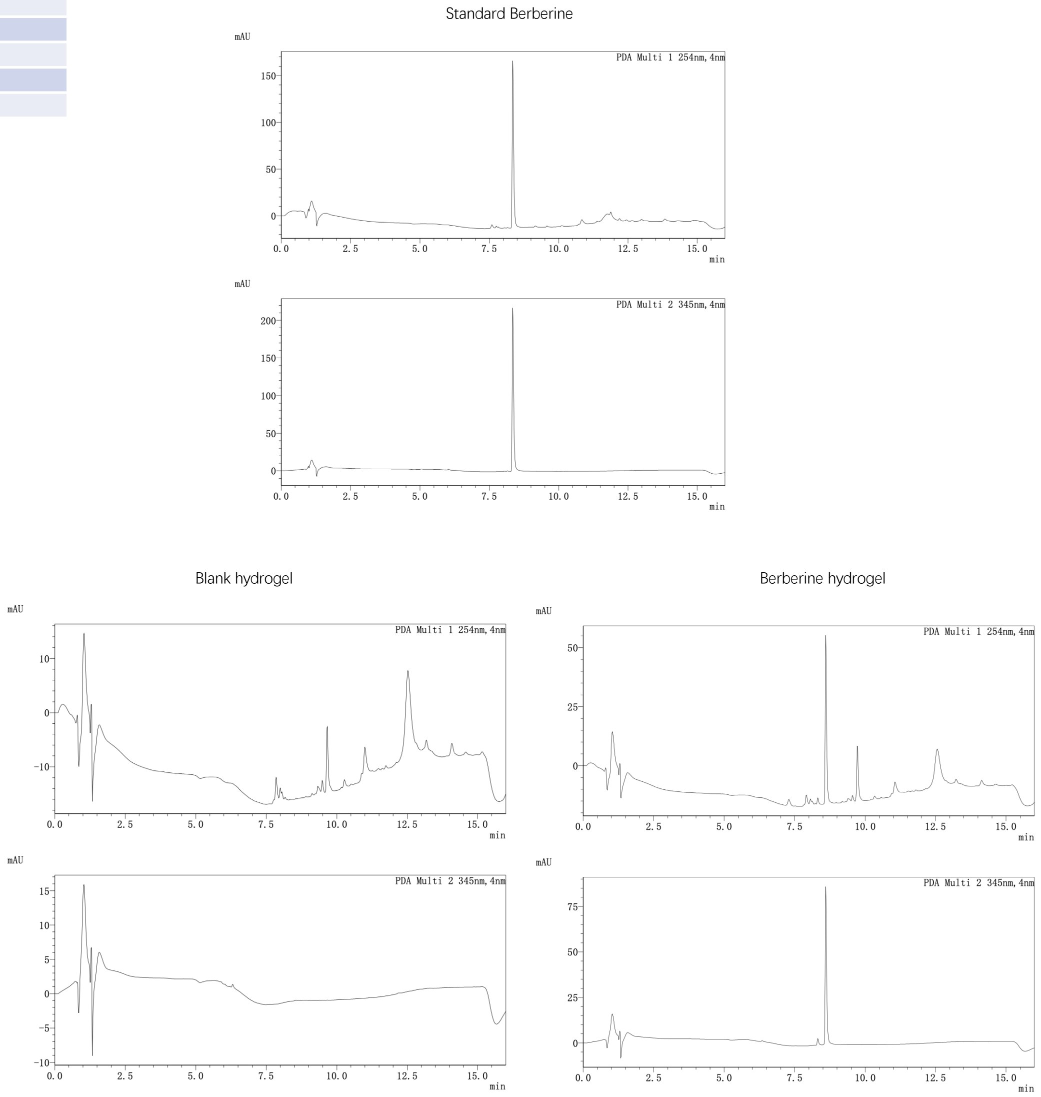

Figure S2

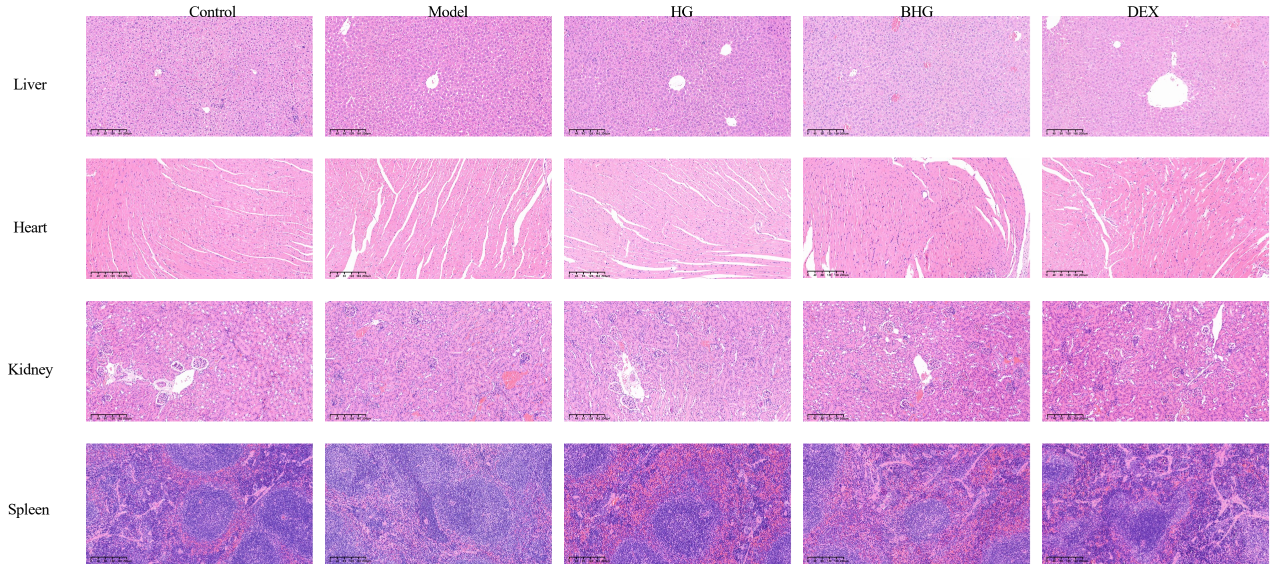

Supplement: Supplementary file 1 [file DataSheet1.pdf]
